# Whisky-Doctors

**Published:** 1860-06

**Authors:** 


### WHISKY-DOCTORS.

A CONSIDERABLE number of persons applying to us for the treatment of throat and lung affections, in answer to the inquiry, "What have you been doing for your ailment," reply, "Using Bourbon whisky:" nine times in ten adding, "It seemed to do good for a while, but has not effected any permanent benefit." Very many persons troubled with a tickling in the throat or annoying hawking or hemming, as a consequence of a disorder of the stomach, evidenced by a changeable or indifferent appetite, bad taste in the mouth of mornings, cold feet, or general chilliness, have been "advised to take a little whisky at meals." The very fact of their seeking further counsel after trying the whisky treatment, is conclusive of its inefficiency. In nearly all the cases, especially from New-England, the practice has been adopted by the "advice of the family physician." One of the best American surgeons known to us, is the most inveterate liquor-drinker. Loving it himself, it was a standard item of commendation to a great number of his patients. This might be accepted as a proof of the efficiency of whisky as a medicine, except for the fact that for the last few years the community have lost confidence in him, and he is no longer considered as A No. 1 among his brethren.

Some physicians have a practice of attempting to make a good first impression by "bolstering" up their patients at once with the various preparations of alcohol or opium, so as to get a good report started. "Why! as soon as Dr. Blank came to see me, I began to get better, even from the first dose of medicine." But when the inevitable death takes place, it is comparatively easy to find a plausible reason for the result, in a sudden change of the weather, in an unfortunate cold, or an error of diet. It is not believed that among regularly-educated practitioners, there is one such in five hundred; but there are such, and it is well for the intelligent reader to be on his guard against employing a man who is not slow to advise whisky, or gin, or beer, or any other alcoholic material, as a daily medicine, and for the very sufficient reason:

The "benefits" arising from the daily use of any thing that can intoxicate is always factitious, unreal, and deceptive, and sooner or later, the cheat will be found out, by the system not only failing to be kept up, but going down to a point lower than that from which it started, with the attendant ill results of its greater inability to rise, and its greater inability to repel the attacks of disease or the ill effects of deleterious agencies.

It is the very nature of alcohol, in all its forms, to goad, and not to strengthen; as it is of opium to blunt and not to eradicate; neither of them ever cured, or aided to cure, any human ailment, except so far as they gave nature time to rally her forces; hence, beyond one or two administrations or "doses," in any given case, their use is mischievous; for, while their immediate effect is deceptive and unsubstantial, the secondary tendency is always, and under all circumstances, to aggravate the malady and to increase the chances against restoration.

It is not contended, at least at this time, that neither opium nor alcohol in any form ought ever to be used as medicines, but it is asserted without any fear of disproof, that the physician who never prescribes or takes either, will be the most successful man as to promptness and permanency and frequency of cure.

It is with these views that two articles have been read in the April number of the *American Gazette*, whose able and veteran editor stands among the highest in the allopathic ranks, which good and wise men will not fail to regret. One is a philippic against Dr. Hiram Cox, of Cincinnati, whose efforts, in an official capacity, to present to the people demonstrative evidence of shameless and perfectly murderous adulterations by the liquor trade certainly merit the countenance and commendation and respect of every benevolent man.

In the same number, which attempts to bring ridicule on Dr. Cox, there is found an indorsement of some body's whisky, all the way from Philadelphia, as the real, original, identical "Jacobs," as being pure as the dew-drop, and "as the very best thing for the sick." This "celebrated" whisky is commended by the editor, on the very conclusive and absolutely irresistible ground that the maker of it stands "vouching that nothing but the genuine pure article" will ever be offered for sale. So will the milkman within five minutes from a "swill-dairy," vouch, nay assert his willingness to make his "affidavy" that he sells pure Orange county milk, and as further proof points to the name of "Orange" in gilt and gold on one end of his cart, and a cow with a long tail on the other, exclaiming, with the conscious pride of argumentative strength: "Don't yer know that it's only stump-tail cows what gives swill milk?" In addition, the official opinions of four expert chemists are paraded to give weight to the whisky; and in close proximity to their names is read: "Such whisky is in many cases of disease, a nutritious and wholesome stimulant." But whether said chemists say these identical words, is not stated; even if they did, their sentiments must be taken with some allowance, for some of these sponsors for the superior excellence of Philadelphia whiskey have "aforetime" stood god-fathers for lager beer, German gin, under the name of "Schiedam Schnapps," and swill-milk; that "lager," in any takable quantity, could not intoxicate, and that there was not such a tremendous sight of difference between swill-milk and other ordinary kinds. It seem to us that Wolfe's gin was chemically analyzed and bepraised by some of these men; and the professional opinions which they have given of the hurtless nature of the contents of patent medicines which have been submitted to their analysis for the last few years, would fill a volume. Does any body with a single mite of sense left, suppose that a patent medicine compounder would put the real constituents of his "stuff" in the sample which he furnished the chemist for analysis? Would he not be a "born fool" not to keep out of "that bottle" at least, all the corrosive sublimate, prussic acid, sugar of lead, copperas, and strychnine which was common to the articles "on sale"? It is greatly to be regretted that scientific men should for pay, in money or soft sawder, lend their influence to simpletons and knaves, to the risk of the health and life itself of the community at large.

The assertion that Mr. Thingumbob's Philadelphia whisky is "nutritious," and "good for the sick," draws rather strong on common-sense; but money is the stronger—so down it goes! This Philadelphia whisky is said to be "celebrated." We never heard of it before, or in our multitude of exchanges came across its name once; but on the day we read of it, we heard of a "Philadelphia fact" of some "celebrity," and from one of the denizens thereof, that within the memory of the present generation, it has never been known that so many persons have died suddenly as during the last winter in the City of Brotherly Love; that a most extraordinary mortality has been observed to prevail during the last eighteen months among business men and others of the "fast" class, between the ages of forty-five and fifty-five. They herded together pretty much. They dined, and drank, and played, and champaigned, and terrapined, and lobstered with each other by turns. They were very proper men. Nobody ever saw them drunk!

> "They were nae fou'
> But just had plenty."

Now if the Philadelphia man's whisky was so particularly good, it must have come to their knowledge and patronage, and putting the two together, it is perfectly plain as to the manner in which the apple got into the dumpling and the milk into the cocoa-nut. "Vivas," long and loud to chemical experts and accommodating editorial doctors, to Philadelphia whisky and humbug!

